# CD4^+^ and CD8^+^ T Cell Activation Are Associated with HIV DNA in Resting CD4^+^ T Cells

**DOI:** 10.1371/journal.pone.0110731

**Published:** 2014-10-23

**Authors:** Leslie R. Cockerham, Janet D. Siliciano, Elizabeth Sinclair, Una O'Doherty, Sarah Palmer, Steven A. Yukl, Matt C. Strain, Nicolas Chomont, Frederick M. Hecht, Robert F. Siliciano, Douglas D. Richman, Steven G. Deeks

**Affiliations:** 1 HIV/AIDS Division, San Francisco General Hospital, University of California San Francisco, San Francisco, California, United States of America; 2 Department of Medicine, Johns Hopkins University School of Medicine, Baltimore, Maryland, United States of America; 3 Division of Experimental Medicine, University of California San Francisco, San Francisco, California, United States of America; 4 University of Pennsylvania, Philadelphia, Pennsylvania, United States of America; 5 Westmead Millennium Institute for Medical Research, Westmead, Australia; 6 University of Sydney, Sydney, Australia; 7 Karolinska Institutet, Stockholm, Sweden; 8 San Francisco VA Medical Center, San Francisco, California, United States of America; 9 Veterans Affairs San Diego Healthcare System, San Diego, California, United States of America; 10 University of California San Diego, La Jolla, California, United States of America; 11 Vaccine and Gene Therapy Institute Florida, Port St. Lucie, Florida, United States of America; 12 Howard Hughes Medical Institute, Baltimore, Maryland, United States of America; New York University, United States of America

## Abstract

The association between the host immune environment and the size of the HIV reservoir during effective antiretroviral therapy is not clear. Progress has also been limited by the lack of a well-accepted assay for quantifying HIV during therapy. We examined the association between multiple measurements of HIV and T cell activation (as defined by markers including CD38, HLA-DR, CCR5 and PD-1) in 30 antiretroviral-treated HIV-infected adults. We found a consistent association between the frequency of CD4^+^ and CD8^+^ T cells expressing HLA-DR and the frequency of resting CD4+ T cells containing HIV DNA. This study highlights the need to further examine this relationship and to better characterize the biology of markers commonly used in HIV studies. These results may also have implications for reactivation strategies.

## Introduction

The use of antiretroviral therapy (ART) has resulted in a marked decline in the morbidity and mortality associated with HIV infection. However, ART does not fully restore health and immune function, as many HIV-infected individuals exhibit evidence of persistent inflammation and immune dysfunction, even after years of effective therapy [1], [2]. ART also fails to fully eradicate HIV, given the establishment of an infected cell population that harbors replication-competent virus known as the viral reservoir [3], [4]. The mechanisms that establish and maintain this reservoir are the focus of intense research.

Among antiretroviral-untreated individuals, there is a strong and consistent association between markers of T cell activation and dysfunction (as defined by markers including CD38, HLA-DR, CCR5 and PD-1) and plasma HIV RNA levels. HIV replication clearly contributes to these immunologic abnormalities, but many of these immunologic abnormalities could also contribute to higher rates of viral replication, as activated CD4+ T cells are the preferred targets for HIV infection. Higher frequencies of proliferating cells (Ki67+) and PD-1+ cells also correlate with higher levels of HIV replication in the absence of therapy [5], [6].

The association between immune activation/dysfunction and total HIV levels during effective ART is less clear. While some studies have found a positive association between the levels of T cell proliferation/activation and levels of cell-associated HIV DNA or RNA [7], [8] other studies have failed to confirm this finding [9]–[11]. A modest correlation between levels of proviral HIV DNA during ART with the frequency of CD4^+^ T cells expressing PD-1 has also been seen in one study [8].

Defining the association between host environment and the size and distribution of the viral population during ART has also been limited in large part by the lack of a well-accepted assay for quantifying HIV levels during effective therapy. In a study of 30 ART-treated adults, we found that most measures of HIV DNA (with the exception of integrated HIV DNA in PBMCs, ρ = 0.7, P = 0.0008) did not correlate with the frequency of cells that can be induced to produce replication-competent virus, as measured by the quantitative viral outgrowth assay (Q-VOA) [12]. Although many have argued that Q-VOA should be the gold standard, a recent study found that a subset of non-induced proviruses is replication-competent and inducible with repeated stimulation [13]. Therefore, the Q-VOA likely underestimates the reservoir size and the true size of the reservoir likely lies somewhere in between the measurements of PCR-based methods and the Q-VOA. Consequently, a more integrative approach that incorporates data from multiple assays may be needed to fully characterize the virus population during ART.

Given these gaps in knowledge, we examined the association between HIV levels as measured in our recent study and markers of T cell activation and function. Our primary objective was to determine if the frequency of activated cells correlated with measures of HIV in this well-characterized cohort. We found a strong association between the frequency of CD4^+^ and CD8^+^ T cells expressing HLA-DR and the frequency of resting CD4+ T cells containing HIV DNA. This study highlights the need to further examine this relationship and to better characterize the biology of markers commonly used in HIV studies. Furthermore, these results may have implications for reactivation strategies.

## Methods

### Ethics Statement

All subjects provided written informed consent. This study was approved by the Committee on Human Research, the institutional review board of the University of California, San Francisco.

### Study Population

Participants were identified from the University of California, San Francisco (UCSF) Options and SCOPE cohorts, as previously described [12]. Briefly, subjects were eligible for enrollment if they were: 1) HIV-infected; 2) on ART with plasma RNA<40 copies/mL for ≥36 months; and 3) had> 350 CD4^+^ T cells/µl for ≥6 months. Subjects were excluded if they had been hospitalized or had received systemic antibiotics, a vaccination, or any immunomodulatory drug (including maraviroc) in the preceding 16 weeks.

### Measurements of HIV

All individuals underwent a large volume blood draw (220 mL) and up to 10 different measurements of HIV persistence were performed as previously described (Table 1) [12]. Briefly, 180 mL of the sample were used to isolate peripheral blood mononuclear cells (PBMCs) and resting CD4^+^ T cells. Resting CD4^+^ T cells were purified by negative selection using biotin-conjugated antibodies to CD25, CD69 and HLA-DR and anti-biotin microbeads (Miltenyi, San Diego, CA, USA). The viral outgrowth assay was performed using resting CD4^+^ T cells as previously described [12], [14] (See Methods S1 for full methods of each assay). Aliquots of PBMCs and resting CD4^+^ T cells were assayed for total HIV DNA and 2-long terminal repeat (2-LTR) circles using droplet digital PCR (ddPCR) [15] and for integrated HIV DNA using Alu PCR [16]–[19]. A single copy assay (SCA) for plasma virus was also performed [20]–[22]. Of note, not all assays were performed for every subject due to cell availability. A subset of subjects underwent rectosigmoid biopsies and quantitative PCR was used to measure HIV-1 DNA and RNA in rectal cells [23], [24].

**Table 1:** Comparison of HIV reservoir assays performed.

Assay	Source types analyzed	Viral species detected	Replication competent or incompetent?	Advantages	Disadvantages
Quantitative viral outgrowth assay	Resting CD4^+^ T cells	HIV-1 Gag protein (ELISA for p24)	Replication competent only	Quantifies minimum number of infected cells with replication competent virus	Costly, labor-intensive, large volume blood draw required, small dynamic range
Droplet digital PCR for HIV-1 DNA	PBMCs or CD4^+^ T cells	Total HIV-1 DNA, 2-LTR circles	Replication competent and incompetent	May perform better at low copy numbers and manage target sequence variation better than other PCR-based methods	Does not differentiate between replication competent and defective viral strains
Alu PCR for integrated HIV-1 DNA	PBMCs or CD4^+^ T cells	Integrated HIV-1 DNA	Replication competent and incompetent	Correlates best with the viral outgrowth assay	Does not differentiate between replication competent and defective viral strains, more technically difficult than other PCR based methods
Quantitative PCR for HIV-1 DNA and RNA in rectal CD4+ T cells	Total gut cells or sorted CD4+ T cells from rectal biopsies	Total HIV-1 DNA or RNA	Replication competent and incompetent	Measures HIV levels in the gut, where the virus is likely concentrated	Does not differentiate between replication competent and defective viral strains, requires subjects to undergo gut biopsies
Single copy quantitative PCR assay on plasma virus	Plasma	Plasma HIV-1 RNA	Replication competent and incompetent	Quantifies HIV RNA in plasma under the limit of conventional assays	Does not provide a quantification of the number of latently infected cells

### T-Cell Immunophenotyping and Cytokine Staining

The remaining blood was used to isolate PBMCs using previously described methods [25]. PBMCs were thawed, washed and stained with LIVE/DEAD Fixable Aqua Dead Cell Stain Kit (Invitrogen), and then stained with the following fluorescently-conjugated monoclonal antibodies: CD8-QDOT 605 and CD4-PE-Texas Red (Invitrogen, Grand Island, NY, USA); CD3-Pacific Blue, CCR5-PE-Cy5, CD38-PE, HLA-DR-FITC, PD-1 Alexa Fluor 647, CD45RA-PE-Cy7 (BD Biosciences, San Jose, CA, USA); and CCR7-APCeFluor 780 (eBioscience, San Diego, CA, USA). Naïve and memory T cell subsets were defined by quadrant gating with FMO controls on a CD45RA versus CCR7 plot.

For cytokine staining, PBMCs were stimulated for 18–22 h at 37°C with overlapping peptide pools corresponding to HIV-1 Con B Gag peptides (NIH 8117) in the presence of 0.5 µg/mL Brefeldin A and 05 µg/mL Monensin (Sigma-Aldrich, St. Louis, MO, USA). A control well with no stimulation was run in parallel for each sample. Cells were washed and stained with AARD, fixed, and permeabilized for intracellular staining with antibodies against CD3-Pacific Blue, IFN**γ**-FITC, IL-2-PE (BD BioSciences), CD4-PE Texas Red, and CD8-QDot 605 (Invitrogen). Data were compensated and analyzed using FlowJo V9 (TreeStar, Ashland, OR, USA).

### Statistical Methods

The Wilcoxon rank-sum test was used to compare baseline characteristics between those treated during early versus chronic infection. Spearman rank correlation coefficients were calculated to assess associations between host immune responses and measurements of HIV. All statistical analyses were performed using STATA/SE 12 (Stata Corp, College Station, TX, USA).

## Results

### Study population

Thirty individuals were studied, 20 subjects who began ART during chronic infection and 10 who started treatment within 6 months of estimated infection (Table 2). Those treated in acute/early infection were younger than those treated in chronic infection (mean age 47.8 vs. 55.9, P = 0.03). Subjects were also predominantly male and white/non-hispanic. The amount of time with plasma viral load <75 copies/mL was higher in the chronically treated group, although this difference was not statistically significant. Current CD4 counts were similar between groups; however, as expected, those treated in acute/early infection had significantly higher nadir CD4 counts compared to the chronically treated group (P = 0.002).

**Table 2:** Baseline characteristics of the subjects.

	Acute (n = 10)	Chronic (n = 20)	P-value^a^
Age	47.8 (9.3)	55.9 (8.7)	0.03
Male	10 (100)	20^b^ (100)	
White	9 (90)	14 (70)	0.23
Suppression time (years)	5.8 (2.5)	8.0 (4.2)	0.15
Current CD4	726 (287)	672 (144)	0.86
Nadir CD4	411 (159)	203(138)	0.002

Numbers are mean (SD) or n (%).

a The Wilcoxon rank-sum test was used to compare baseline characteristics between those treated early versus those treated during chronic infection.

b Two individuals identified as Male-to-Female transgender.

### Association between T cell activation and HIV levels

**Figure 1:**
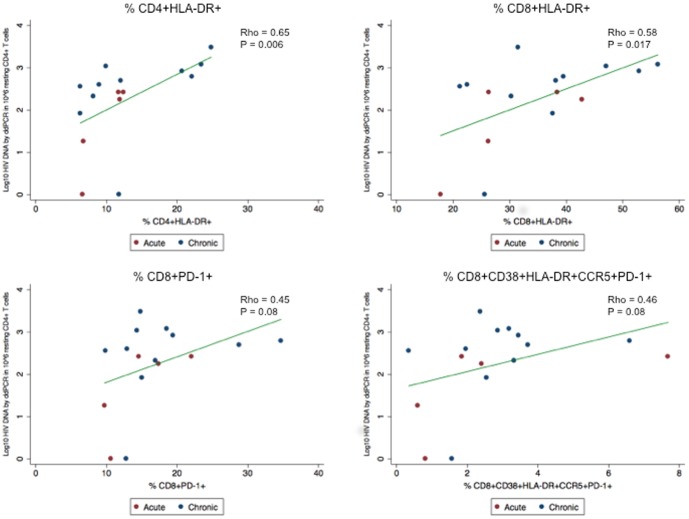
Markers of T cell activation are associated with levels of HIV DNA in resting CD4^+^ T cells. Scatter plots of the association between markers of T cell activation and Log_10_ copies of HIV DNA in 10^6^ resting CD4^+^ T cells as measured by droplet digital PCR (ddPCR) are shown. Linear regression lines are also shown. Of note, n = 16 as ddPCR in resting CD4^+^ T cells was not performed for every subject due to cell availability.

To study the relationship between T cell activation and HIV levels, we assessed for correlations between the frequencies of “activated” cells and multiple different measures of HIV. The most consistent and strongest association was between the frequencies of CD4^+^ or CD8^+^ T cells expressing HLA-DR and HIV DNA levels as measured by ddPCR in resting CD4^+^ T cells (CD4^+^: ρ = 0.65, P = 0.006; CD8^+^: ρ = 0.58, P = 0.017) (Figure 1, Table 3). Similar correlations were also observed when activation was defined by the percentage of CD4^+^ or CD8^+^ T cells co-expressing CD38 and HLA-DR; however, these correlations were mainly driven by HLA-DR, as no correlations were observed with CD38 alone. The association between HIV DNA and HLA-DR expression was strongest in the central memory (CM) and effector memory (EM) subsets of CD4^+^ T cells (%DR^+^ CM: ρ = 0.59, P = 0.015; %DR^+^ EM: ρ = 0.64, P = 0.007), whereas in CD8^+^ T cells the correlations were more similar between the different T cell subsets (ρ = 0.33–0.59), but were only statistically significant in the central and effector memory CD8^+^ populations ((%DR^+^ CM: ρ = 0.59, P = 0.015; %DR^+^ EM: ρ = 0.51, P = 0.04). Of note, this relationship between the frequency of activated CD4^+^ and CD8^+^ T cells and the levels of HIV DNA in resting CD4^+^ T cells remained consistent even when individuals treated in acute versus chronic infection were analyzed separately (data not shown).

**Table 3:** Correlations coefficients^a^ for HIV measurements and frequency of T cells expressing activation markers CD38 and HLA-DR.

Assay	CD4^+^38^+^DR^+^	P-value^b^	CD4^+^DR^+^	P-value	CD8^+^38^+^DR^+^	P-value	CD8^+^DR^+^	P-value
Viral outgrowth assay in resting CD4^+^ T cells (n = 30)	0.23	0.22	0.12	0.53	0.10	0.61	−0.02	0.92
Droplet digital PCR for total HIV-1 DNA in PBMCs (n = 30)	0.07	0.73	0.11	0.56	0.25	0.18	**0.36**	**0.049**
Droplet digital PCR for total HIV-1 DNA in resting CD4^+^ T cells (n = 16)	**0.64**	**0.007**	**0.65**	**0.006**	**0.51**	**0.044**	**0.58**	**0.017**
Naïve T cells	0.35	0.18	0.31	0.24	0.33	0.21	0.33	0.22
Central memory	**0.60**	**0.013**	**0.59**	**0.015**	0.33	0.21	**0.59**	**0.015**
Effector memory	**0.57**	**0.022**	**0.64**	**0.007**	0.26	0.32	**0.51**	**0.044**
Terminally differentiated	0.29	0.28	0.22	0.42	0.39	0.13	0.45	0.08
Droplet digital PCR for HIV-1 2-LTR DNA in PBMCs (n = 30)	0.28	0.13	0.25	0.18	0.28	0.14	.30	0.11
Droplet digital PCR for HIV-1 2-LTR DNA in resting CD4^+^ T cells (n = 16)	0.20	0.46	0.28	0.29	0.44	0.09	**0.57**	**0.025**
Alu PCR for integrated HIV-1 DNA in PBMCs (n = 19)	0.27	0.27	0.20	0.41	0.13	0.61	−0.02	0.93
Alu PCR for integrated HIV-1 DNA in resting CD4^+^ T cells (n = 15)	0.22	0.43	0.05	0.85	0.06	0.84	−0.09	0.73
Quantitative PCR for HIV-1 DNA in rectal CD4+ T cells (n = 19)	−0.14	0.56	−0.18	0.45	−0.22	0.37	−0.18	0.47
Quantitative PCR for HIV-1 RNA in rectal CD4+ T cells (n = 19)	−0.16	0.52	−0.19	0.44	−0.25	0.31	−0.19	0.44
Single copy PCR assay^c^ on plasma virus (n = 30)	−0.26	0.16	−0.17	0.36	**−0.52**	**0.003**	**−0.50**	**0.005**

a Spearman correlation coefficients are shown.

b Correlation coefficients and the corresponding p-values are bolded if p<0.05.

c Note, half the limit of detection was used for values that were less than the limit of detection.

A correlation between HLA-DR expression in CD8^+^ T cells and total HIV DNA levels measured by ddPCR in total PBMCs was also seen, but was more modest (ρ = 0.36, P = 0.049) and this association was not seen in CD4^+^ T cells. A modest correlation between 2-LTR circles measured in resting CD4^+^ T cells and PBMCs with the frequency of HLA-DR expression in CD4^+^ and CD8^+^ T cells was also noted. However this association was only statistically significant between % CD8^+^ HLA-DR^+^ T cells and the amount of 2-LTR circles in resting CD4^+^ T cells. There were no consistent associations between the expression of activation markers on CD4^+^ or CD8^+^ T cells and integrated DNA measured by Alu PCR in either resting CD4^+^ T cells or PBMCs. HIV RNA and DNA levels in rectal tissue also did not correlate with either HLA-DR expression alone or with CD38. A statistically significant negative correlation was seen between measurements of plasma viremia by the single copy assay and activation in CD8^+^ T cells. Lastly, no consistent associations were seen between activation markers and measurement of HIV by the viral outgrowth assay.

A moderate correlation was also seen between the frequencies of resting CD4^+^ T cells containing HIV DNA by ddPCR and either the expression of PD-1 or all markers (CD38^+^HLA-DR^+^CCR5^+^PD1^+^) on CD8+ T cells (PD-1: ρ = 0.45, P = 0.08; All: ρ = 0.46, P = 0.08) (Figure 1) although it did not reach statistical significance. A more modest association between CD8^+^PD1^+^ T cells and total HIV DNA in PBMCs was also noted (ρ = 0.37, P = 0.04). This relationship was not seen in CD4^+^ T cells.

### Association between frequency of HIV-specific T cells and HIV levels

**Figure 2:**
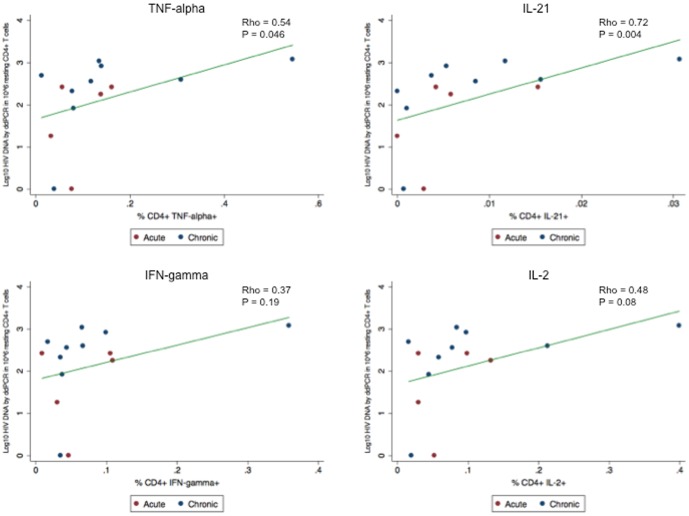
The frequency of HIV-specific CD4+ T cells is associated with HIV DNA levels in resting CD4^+^ T cells. Scatter plot of the association between the frequency of HIV-specific CD4+ T cells expressing TNF- α, IL-21, IFN- γ, and IL-2, and Log_10_ copies of HIV DNA as measured by droplet digital PCR (ddPCR). Linear regression lines are also shown.

HIV-specific CD4^+^ T cells expressing TNF-α and IL-21 also correlated positively with total HIV DNA in resting CD4^+^ T cells by ddPCR, although the frequency of cells expressing IL-21 was low (TNF-α: ρ = 0.54, P = 0.046; IL-21: ρ = 0.72, P = 0.004) (Figure 2). Similar non-significant trends were observed with HIV-specific CD4^+^ T cells expressing IL-2 and IFN-γ (IL-2: ρ = 0.48, P = 0.08; IFN-γ: ρ = 0.37, P = 0.19). Although there was notable variation in the frequency of HIV-specific CD4+ T cells for similar HIV DNA values, the 2 subjects with undetectable HIV DNA had consistently low frequencies of HIV-specific CD4^+^ T cells and the subject with consistently high frequencies of HIV-specific CD4^+^ T had one of the highest levels of HIV DNA. These relationships were not observed in HIV-specific CD8^+^ T cells.

## Discussion

Identifying how the host immune environment might shape the size and distribution of HIV-infected cell populations during long-term ART may provide insights into how to cure HIV infection. Leveraging an extensively characterized cohort of well-treated HIV-infected adults [12], we sought to determine the major immune correlates of HIV levels in adults who received ART during either early or late/chronic infection. The most consistent association was between the frequency of CD4^+^ and CD8^+^ T cells expressing HLA-DR and the frequency of resting CD4+ T cells containing HIV DNA as measured by ddPCR. No correlations were noted when looking at CD38 alone, a finding that is consistent with a previous study that did not show an association between activation markers and the frequency of CD4^+^ T cells containing proviral DNA, but only looked at CD38 expression [10]. This may be due, at least partly, to the fact that CD38 is expressed not only on activated cells, but also on naïve T cells [26]. These findings are also consistent with a recent study by Murray, and colleagues [27]. This relationship with HLA-DR expression was consistent between those treated early in infection and those treated in chronic infection.

The strong association between HIV DNA and the frequency of CD4^+^ T cells expressing HLA-DR raises the question as to whether HLA-DR+ cells may represent a preferential reservoir for the virus. In our study, however the strongest associations were seen with HIV DNA measured in resting CD4^+^ T cells, which are HLA-DR^-^ and therefore this hypothesis cannot be tested with this study. If this hypothesis were true, however, one explanation would be that as HLA-DR is upregulated during T cell activation following T cell receptor engagement, its expression may identify activated memory CD4^+^ T cells, which are the preferred target for replicating virus. Another perhaps more likely possibility is that HLA-DR expression reflects in part antigen-independent, homeostatic proliferation. Proliferating T cells also express HLA-DR. In line with this model, IL-7, a regulator of CD4 T cell homeostasis, transiently increases both HLA-DR and PD-1 expression in CD4 T cells both *in vitro* and *in vivo* in humans [28]–[30]. Taken together, these studies and our observations may suggest that higher levels of HLA-DR expression on T cells may reflect enhanced levels of homeostatic proliferation, a mechanism critically involved in HIV persistence during ART [7], [31].

We also found that HIV-specific CD4^+^ T cells expressing TNF-α and IL-21 correlated positively with total HIV DNA in resting CD4^+^ T cells by ddPCR. It is notable that CD4+ T cells expressing IL-21 corresponded with HIV levels as there is both non-human primate and human data to suggest that follicular helper CD4+ T (T_FH_) cells may be a reservoir for HIV infection [32], [33]. All CD4^+^ T cells that produce IL-21 are not necessarily T_FH_ cells however [34], [35]. An alternative explanation for the relationship is that since HIV-specific CD4^+^ T cells are preferentially infected by HIV [36], an increase in the size of this population would be expected to increase the available targets for HIV infection, thereby contributing to increased reservoir size, regardless of the type of CD4+ T cell. Similar associations between HIV-specific CD4^+^ T cells and HIV levels have been seen in controllers [37] and in ART-treated individuals after a therapeutic HIV vaccination [38]. Interestingly, we did not find any relationship with HIV levels and HIV-specific CD8^+^ T cells. Although one might expect that increased levels of HIV-specific CD8^+^ T cells would lead to increased killing of infected cells and decreased reservoir size, a previous study found that even after virus reactivation, CD8^+^ T cells from ART-treated individuals did not kill HIV-infected CD4^+^ T cells [39].

This study has a few limitations that deserve comment. As a cross-sectional study, it is impossible to comment on causality. Although we have argued that T cell proliferation/activation may have contributed to higher frequency of infected cells, it is also possible that more virus causes more activation and/or proliferation. Furthermore, although a virus may not be replication competent, it may still be capable of producing viral proteins, which may lead to resultant activation. Interventional studies will be necessarily to untangle these associations. Also, as there is no well-accepted assay to measure the total body burden of replication-competent HIV (the “reservoir”), it is unclear as how to best measure virus/host associations. Although PCR-based assays may detect defective virus as well as replication-competent virus, they may not overestimate the size of the latent reservoir as much as previously thought [13]. Droplet digital PCR may have performed well in our analysis given its capacity to manage target sequence variation and the fact that it may be more precise for those samples with low copy numbers [15]. If one assumes that CD4^+^ T cells are the main reservoir for HIV, then assays that use isolated CD4+ T cells may also provide increased sensitivity, especially in those individuals with lower viral burden. Finally, much of the virologic measurements were performed in resting memory cells. These cells were selected based in part on the lack of HLA-DR. However, a previous study found that even years after ART, there were as many integrated viral genomes in activated memory cells as there were in the resting memory cell population on a per cell basis [27] suggesting activated cells may contribute to the reservoir. Further studies in which cells are sorted based on HLA-DR and the presence or absence of other activation markers are also now being planned.

Although defining causation in this study is not possible, our study nonetheless provides evidence of the association between markers of T cell activation/proliferation and HIV persistence. It also highlights the need for future studies to further examine this relationship and to better characterize the biology of various markers commonly used in HIV research. Furthermore, it may have implications for reactivation strategies, as reversal of latency, in the absence of effective killing and complete suppression of viral replication, could lead to an increase in reservoir size.

## Supporting Information

Methods S1 **Detailed Methods of HIV Quantification Assays.**
(DOCX)
