# Supplementary material for: CD4+ and CD8+ T Cell Activation Are Associated with HIV DNA in Resting CD4+ T Cells
Source: PLoS One. 2014 Oct 23;9(10):e110731. doi: 10.1371/journal.pone.0110731 (PMC4207702; doi:10.1371/journal.pone.0110731)
Supplement: Methods S1 — Detailed Methods of HIV Quantification Assays. (DOCX) [file pone.0110731.s001.docx]

**Methods S1:** Detailed Methods of HIV Quantification Assays

**Resting CD4+ T cell isolation.** A two step protocol using biotinylated antibodies and anti-biotin magnetic beads was performed to isolate resting CD4+ T cells by negative depletion from PBMC. Briefly, non-CD4+ T cells were labeled first with a cocktail of biotin-conjugated monoclonal antibodies followed by incubation with anti-biotin-conjugated magnetic microbeads (CD4+ T cell Isolation Kit II; Miltenyi). Unwanted cells were then removed using a LS MACS Column with the MACS Separator magnet (Miltenyi). Activated CD4+ T lymphocytes were then removed from the total CD4+ T cell population by labeling unwanted cells with biotin-conjugated antibodies to CD25, CD69, and HLA-DR and anti-biotin microbeads (Miltenyi). Labeled cells were again removed using a LS MACS Column with the MACS Separator magnet.

**Viral outgrowth assay.** A limiting dilution virus culture assay was performed as previously described [13] except that purification of resting CD4+ T cells was carried out using a two step magnetic bead depletion protocol as above. Five-fold serial dilutions of highly purified resting CD4+ T cells were stimulated with the mitogen PHA and a ten fold excess of irradiated allogeneic PBMC. This stimulation induces global T cell activation which reverses latency at least in a fraction of cells carrying integrated HIV-1 genomes. The release of replication-competent virus was detected by adding CD4+ lymphoblasts from HIV-1-negative donors. The virus replicates in these cells and after 2–3 weeks of culture, supernatants contain sufficient levels of virus that an ELISA assay for HIV-1 p24 antigen can be used to detect positive wells. CD8+ T cells, which can suppress viral replication, were depleted from the lympho- blasts that were added to the culture assay. The frequency of HIV- 1 infected cells was determined by a maximum likelihood method [13].

**Droplet digital PCR for HIV-1 DNA**. The frequency of HIV-1 DNA was measured by droplet digital PCR, following published methods [15]. Cellular DNA was extracted using a Qiagen DNA Blood Midi Kit, following the manufacturer’s protocol. The DNA concentration was estimated from the A260/A280 absorptivity ratio using a NanoDrop 2000 spectrophotometer (Thermo Scientific), concentrated by ethanol precipitation if necessary to achieve a minimum concentration of 150 ng /µL, and digested with BSAJ-I (New England Biolabs) at 60**°**C for 1 hour. Primers (mf299 and mf302) and a fluorescent hydrolysis probe (mf348) that match conserved regions of HIV-1 pol were used in triplicate PCR reactions. In a separate reaction, templates were diluted 10-fold and a primer/probe set that recognizes human RNAse P (RPP30) was used for host genomic DNA quantification. The PCR reaction mixture was loaded into the Bio- Rad QX-100 emulsification device, and droplets were formed following the manufacturer’s instructions. The emulsified droplets were transferred to a 96-well reaction plate, sealed with a Bio-Rad heated plat sealer, and transferred to an Applied Biosystems GeneAmp 9700 thermal cycler for amplification. Each reaction consisted of a 20 μL solution containing 10 μL of ddPCR Probe Supermix, 900 nM primers, 250 nM probe, and either 1 µg of template DNA (for HIV-1 pol) or 100 ng of template DNA (for RPP30). Cycling conditions were the following: 10 minutes at 95**°**C, 40 cycles each consisting of a 30 second denaturation at 94**°**C followed by a 60**°**C extension for 60 seconds, and a final 10 minutes at 98**°**C. After cycling, droplets were stored at 4**°**C in the dark until analysis. Thresholds were set and copy numbers per reaction were computed using Bio-Rad Quantasoft software, version 1.3.2.

**Measurements of integrated HIV-1 in resting CD4+T cells and PBMC.** Integrated HIV-1 DNA was measured in PBMC or purified resting CD4+ T cells using a previously described Alu PCR [15-17]. This assay detects only integrated proviruses because it relies on an initial amplification in which one primer hybridizes with a conserved sequence in *Alu* elements which are present in ~2,000,000 copies in the human genome. The *Alu* primer is paired with an HIV-1 *gag* primer in the first round of amplification. This *Alu-gag* amplification is then followed by a second amplification that targets a sequence in the HIV-1 LTR (R-U5). The level of HIV-1 integration is quantitated by comparing the detection signal to an integration standard curve that correlates cycle thresholds with integration standard copy number. The integration standard was especially produced to contain genomes with integration sites at a variety of distances from *Alu* sites, mimicking the pattern of integration seen in natural infection [80]. DNA was isolated from frozen PBMC and resting CD4+T cell pellets using the Gentra Puregene Cell Kit (Qiagen) following the manufacturer’s instructions. DNA was then diluted to 2 μg/mL and assayed in 42 replicates for integrated HIV-1 proviruses. The first step reaction was performed for 40 cycles at the following conditions: 95**°**C for 15 s, 50**°**C for 15 s and 72**°**C for 3 min 30 s. Simultaneously and on the same plate, 42 reactions with only the *gag* primer were also performed following the same conditions. The product from the first step reaction was then diluted 1:2 into the 2nd step master mix. The second step was performed on an ABI 7500 FAST instrument following the FAST protocol (95**°**C for 0:03 and 60**°**C for 0:30) for 50 cycles. The cycle threshold values are then used to calculate an integration value using a standard curve as described [18]. In samples with low levels of integrated HIV-1 DNA (with integration detectable in <30% of wells), the percent positive method for calculation was utilized. In these cases, a cutoff value was generated by subtracting two standard deviations from the average of the cycle threshold values for PCR reactions with only the *gag* primer present in the first step. Any wells with a cycle threshold less than this cutoff value were considered ‘‘positive’’ signals. The percent of positive signals is calculated by dividing the wells counted by the total number of wells assayed (containing both the *Alu* and *gag* primers). A standard curve [18] showing a linear relationship between the copies of integrated HIV-1 and the percent of positive wells was used to calculate copy number in several of these samples. Occasionally, samples had too little DNA to do 42 replicates.

**Single copy assay for residual viremia.** The concentration of HIV-1 RNA in plasma was determined as previously described [19-21]. To control for recovery of HIV-1, each plasma sample is spiked with 30,000 copies of an internal virion standard derived from an unrelated retrovirus, the replication competent avian sarcoma-leukosis retrovirus vector RCAS BP(A). For samples expected to have <50 copies of HIV-1 RNA/ml, 8 ml of plasma were used. Total RNA was extracted from the pellet using proteinase K and guanidinium isothiocyanate and used as a template for reverse transcription and real-time PCR of a small (79 bp), highly conserved, coding sequence in HIV-1 *gag*. Using this primer and probe set, HIV-1 RNA can be amplified from about 80% of those infected with HIV-1 subtype B. PCR products are detected using fluorescent probes. For each plasma sample, 3 independent reactions were run with HIV-1 gag-specific primers and two with RCAS BP(A) gag-specific primers. The quantity of HIV-1 RNA in the original plasma sample was calculated from a standard curve generated for each assay using serial dilutions of in vitro transcripts of the same region of gag. Control experiments have shown that the recovery of HIV-1 RNA and RCAS BP(A) RNA was comparable and independent of the original plasma volume used in the range of 1–8 ml.

**Assays for HIV-1 DNA and RNA in GALT.** Rectal biopsy samples were separated into single cells using a modification of a published method [22, 23]. Briefly, biopsies were subjected to three rounds of collagenase digestion, mechanical disruption (by passing through a blunt 16 gauge needle), clarification (by passing through a 70 µm cell strainer), and washing. The three aliquots of strained and washed cells were then combined, counted, and aliquoted for flow cytometry and nucleic acid extraction. For extraction, cells were lysed using 1 ml of Qiazol reagent (Qiagen), followed by 15 min centrifugation at 12,000 g at 4°C. RNA was extracted from the aqueous layer using the miRNeasy Mini kit (Qiagen) with on-column DNAase treatment (Qiagen RNase-Free DNase Set) and eluted in 30 µl of RNase-free water. DNA was extracted from the organic layer using 0.5 ml of back extraction buffer (4 M guanidine thiocyanate, 50 mM Na citrate, and 1 M Trizma base), followed by precipitation with 500 µl of isopropanol, 2 washes with 75% ethanol, and resuspension in 100 µl of EB buffer (Qiagen). RNA and DNA concentrations were measured using a ND-1000 spectrophotometer (NanoDrop).

Three replicate samples of 500 ng of DNA from each donor were assayed for total HIV-1 DNA using a modification of a published real time Taqman PCR assay that uses primers and a probe from the LTR region and can detect a single copy of HIV-1 in brain tissue [3]. Primers were F522-43 Kumar (5′ GCC TCA ATA AAG CTT GCC TTG A 3′; HXB2 522–543) and R626-43 Kumar long (5′ GGG CGC CAC TGC TAG AGA 3′; 626–643). The probe, 5′ CCA GAG TCA CAC AAC AGA CGG GCA CA 3′, was dual-labeled with 6-FAM(5′) and Black Hole Quencher BHQ-1(3′). Reaction volume was 50 µl with 25 µl of 2× Gene Expression Master Mix (Applied Biosystems), 10 pmol of each primer, 10 pmol of probe, and 5 µl of DNA (100 ng/µl). Cycling conditions were: 50°C for 2 min, 95°C for 10 min, then cycles of 95°C for 15 s and 59°C for 1 min. External standards (10^5^ to 1) were prepared from DNA extracted from known numbers of 8E5 cells (NIH AIDS Reagent Program), each of which contains one integrated HIV-1 genome per cell.

HIV-1 DNA copy numbers were measured in triplicate and normalized to cellular input, as determined by DNA concentration (assuming 1 µg total DNA corresponds to 160,000 cells). To account for variation in the number of CD4^+^ T cells in different samples, results were further normalized by the percent of all cells that were CD45^+^CD3^+^CD4^+^ (by flow cytometry) and expressed as copies/10^6^ CD4^+^ T cells [22]. This normalization is based on the assumption that most of the HIV-1 DNA in rectal cells is in CD4^+^ T cells. This assumption is based on a recent analysis of sorted rectal cell populations that found ~90% of the HIV-1 DNA to be in CD4+ T cells (S. Yukl, unpublished results).

Three replicates of up to 500 ng of RNA from each sample were assayed for total processive HIV-1 RNA transcripts using primers and probe from the LTR region (as above). Reaction volume was 50 µl with 25 µl of 2× One Step RNA-to-Ct mix (Applied Biosystems), 10 pmol of each primer, 10 pmol of probe, 1.25 µl of 40× RT (Applied Biosystems), and 5 µl of RNA (100 ng/µl). Cycling conditions were: 48°C for 20 min, 95°C for 5 min, then 60 cycles of 9°5C for 15 s and 59°C for 1 min. External standards (2.5 to 2.5×10^5^) of genomic HIV-1 RNA were prepared by extracting the RNA from lab stocks of NL4-3 virions (using the QIAmp Viral RNA Mini Kit) and then quantifying the RNA via replicate measurements using the Abbot Real Time assay.

HIV-1 RNA copy numbers were measured in triplicate and normalized to cellular input into the PCR, as determined by RNA concentration (assuming that 1 ng RNA correspond to 1000 cells), which has been shown to correlate with levels of glyceraldehyde phosphate dehydrogenase (GAPDH) RNA. As an alternative method of normalizing to cell numbers, all RNA samples were assayed for GAPDH in triplicate by qRT-PCR with the conditions above, except with 2.5 µl of 20× GAPDH primer/probe mix [Applied Biosystems] and 3 µl of RNA. To account for variation in the number of CD4^+^ T cells in different samples, HIV-1 RNA copy numbers were further normalized by the percent of all cells that were CD45^+^CD3^+^CD4^+^[22].
